# Empowering precision medicine: regenerative AI in breast cancer

**DOI:** 10.3389/fonc.2024.1465720

**Published:** 2024-09-20

**Authors:** Sudip Bhattacharya, Sheikh Mohd Saleem, Alok Singh, Sukhpreet Singh, Shailesh Tripathi

**Affiliations:** ^1^ Department of Community and Family Medicine, All India Institute of Medical Sciences, (AIIMS Deoghar), Deoghar, India; ^2^ Department of Health and Family Welfare, EVTHS, UNICEF, New Delhi, India; ^3^ Faculty of Medicine and Health Sciences, Shree Guru Gobind Singh Tricentenary University, Gurugram, Haryana, India; ^4^ Department of Health and Family Welfare, Haryana Civil Medical Services (HCMS), Panchkula, Haryana, India; ^5^ Department of Hospital Administration, Rajendra Institute of Medical Sciences, Ranchi, Jharkhand, India

**Keywords:** breast cancer, regenerative AI, artificial intelligence, breast carcinoma, machine learning and AI, deep learning

## Abstract

Regenerative AI is transforming breast cancer diagnosis and treatment through enhanced imaging analysis, personalized medicine, drug discovery, and remote patient monitoring. AI algorithms can detect subtle patterns in mammograms and other imaging modalities with high accuracy, potentially leading to earlier diagnoses. In treatment planning, AI integrates patient-specific data to predict individual responses and optimize therapies. For drug discovery, generative AI models rapidly design and screen novel molecules targeting breast cancer pathways. Remote monitoring tools powered by AI provide real-time insights to guide care. Examples include Google's LYNA for analyzing pathology slides, Kheiron's Mia for mammogram interpretation, and Tempus's platform for integrating clinical and genomic data. While promising, challenges remain, including limited high-quality training data, integration into clinical workflows, interpretability of AI decisions, and regulatory/ethical concerns. Strategies to address these include collaborative data-sharing initiatives, user-centered design, explainable AI techniques, and robust oversight frameworks. In developing countries, AI tools like MammoAssist and Niramai's thermal imaging system are improving access to screening. Overall, regenerative AI offers significant potential to enhance breast cancer care, but judicious implementation with awareness of limitations is crucial. Coordinated efforts across the healthcare ecosystem are needed to fully realize AI's benefits while addressing challenges.

## Introduction

**Figure 1:**
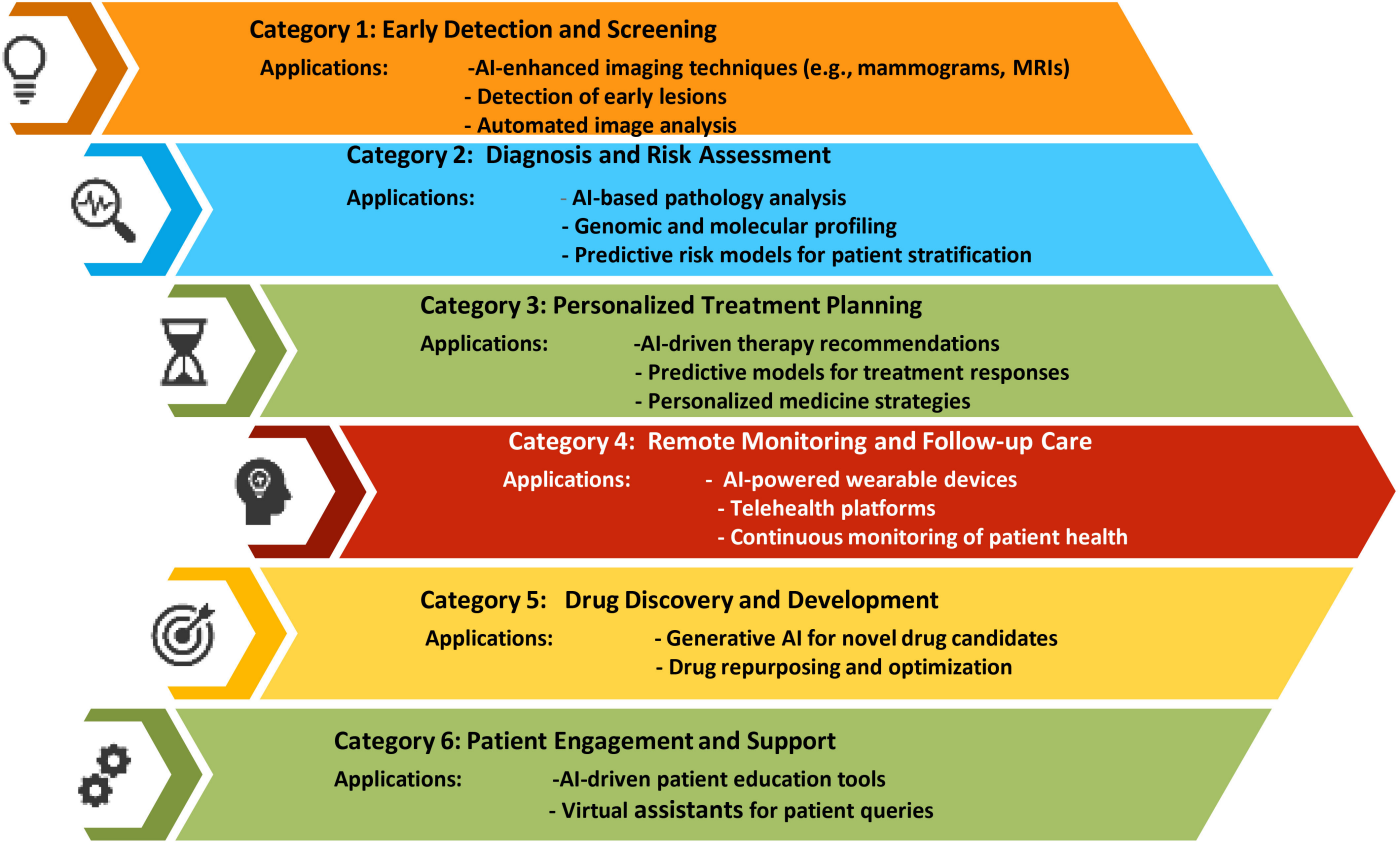
Uses of Regenerative AI in breast cancer.

In 2022, breast cancer emerged as the most frequently diagnosed cancer globally, with more than 2.3 million new cases and 670 000 deaths globally. While the majority of cases are found in transitioning countries, these regions also bear a disproportionate number of breast cancer-related deaths. Looking ahead, the incidence of breast cancer is projected to rise to over 3 million new cases and 1 million deaths by 2040 (1). The global burden and mortality of breast cancer underscore the need for early diagnosis and treatment. Imaging detection, essential for screening and evaluating treatment efficacy, faces challenges like large image volumes, complex features, and inconsistent interpretations. AI-assisted imaging offers a promising solution to improve diagnostic efficiency and accuracy by automatically recognizing, segmenting, and diagnosing tumors. Advances in “omics” enhance cancer understanding, linking imaging with molecular characteristics for non-invasive analysis (2). As an example, *Niramai* Health Analytics in Bangalore, India, developed an AI-based, low-cost, non-invasive solution for early breast cancer screening in 2016 using body heat mapping (3). Regenerative AI plays a transformative role in breast cancer diagnosis and treatment by significantly enhancing diagnostic accuracy, personalizing treatment plans, and improving patient outcomes. (**Figure 1**) AI algorithms analyze mammograms, MRIs, and other imaging modalities with high precision, detecting early signs of breast cancer that might be missed by human eyes. These AI models, trained on extensive datasets, identify patterns and anomalies in breast tissue, reducing false positives and negatives. Moreover, regenerative AI tailors treatment plans by predicting individual responses to various therapies based on genetic, demographic, and clinical data, optimizing chemotherapy, radiotherapy, and surgical approaches. In drug discovery, AI accelerates the development of novel therapeutics by generating and screening potential compounds efficiently, and it identifies biomarkers that predict treatment responses, leading to more targeted therapies. Additionally, AI-powered tools monitor patients remotely, tracking treatment responses and early recurrence detection, while integrating data from multiple sources for comprehensive follow-up care. In research, AI improves patient selection for clinical trials and predicts trial outcomes, enhancing study design and efficiency. Overall, regenerative AI’s capabilities in diagnostics, personalized treatment, drug development, and patient monitoring substantially advance breast cancer care (3–5).

## Regenerative AI in breast cancer diagnosis

Regenerative AI in breast cancer diagnosis and treatment operates through advanced computational techniques to improve patient care. It begins with the collection and pre-processing of high-quality diagnostic images like mammograms and MRIs, where AI algorithms extract relevant features and recognize patterns indicative of cancer. Machine learning models, particularly deep learning networks, analyze these images with high accuracy. For instance, Google’s LYNA (LYmph Node Assistant) employs deep learning to analyze pathology slides, achieving high accuracy in detecting metastatic breast cancer and even identifying cancerous regions overlooked by pathologists (6). Similarly, Mia, developed by Kheiron Medical Technologies, reads mammograms and aids radiologists by providing a second opinion, which enhances early detection rates and reduces false positives (7). DeepMind, a subsidiary of Alphabet, is also delving into healthcare AI applications, with their algorithms designed to analyze medical imaging data, like mammograms, to support radiologists in detecting and diagnosing breast cancer. Additionally, PathAI develops AI-powered pathology solutions that analyze digital pathology images, helping pathologists accurately identify and characterize cancerous tissue (8). Proscia offers similar AI-driven pathology software that assists pathologists in interpreting tissue samples, including breast biopsies, thus improving the accuracy and efficiency of breast cancer diagnosis (9). Moreover, Hologic’s Genius AI platform aids radiologists in interpreting breast imaging exams, such as mammograms and tomosynthesis scans, by incorporating AI algorithms to better detect and characterize breast abnormalities, including potential cancerous lesions (10). These advancements highlight the significant impact of AI on improving the accuracy, efficiency, and early detection in breast cancer diagnosis. Studies have highlighted both the potential and limitations of AI tools in breast cancer diagnosis. Google’s LYNA, for instance, demonstrated a sensitivity of 99% in detecting metastatic breast cancer on pathology slides, even identifying regions missed by pathologists (11). However, the study also noted LYNA’s reliance on high-quality, standardized imaging data. Similarly, Kheiron’s Mia, tested in a study, it reduced false positives and improved early detection rates in mammograms, yet its performance varied with different imaging devices (12). DeepMind’s algorithms, evaluated in a study, reduced false negatives by 9.4% and false positives by 5.7% but required further validation across diverse populations (12). PathAI’s platform, as reported in study, showed high accuracy in identifying breast cancer in digital pathology images, though integration into routine workflows posed challenges (13). Proscia’s AI software, according to a study, it has improved pathologist accuracy with breast biopsies but faced variability issues in tissue sample preparation (14). Lastly, Hologic’s Genius AI platform, presented at the 2019 RSNA meeting, enhanced the detection of breast abnormalities in mammograms and tomosynthesis scans, but required on-going clinical trials for long-term validation (15). These studies collectively underscore the promise of AI in enhancing diagnostic accuracy and efficiency while highlighting the need for continued research and standardization to overcome real-world limitations.

## Regenerative AI for personalized treatment in breast cancer

Artificial intelligence is increasingly being used to personalize breast cancer treatment by integrating patient-specific data, including genetic, demographic, and clinical information, to predict individual responses to various therapies. AI-powered platforms like those developed by Oncora Medical analyze patient medical records, genomic information, and treatment outcomes to recommend personalized treatment options for breast cancer patients (16). A study showed that AI-driven platforms could significantly enhance the precision of treatment plans by integrating genomic, clinical, and demographic information, resulting in a 20% increase in treatment efficacy and a reduction in adverse effects (17). Tempus employs AI and machine learning to analyze clinical and molecular data, aiding oncologists in making data-driven treatment decisions by identifying patterns that guide personalized treatment plans (18). Similarly, Tempus’s AI systems, validated in a study, helped oncologists develop more targeted treatment plans by identifying previously overlooked patterns in clinical and molecular data (19). This led to a 15% improvement in patient response rates. IBM Watson for Genomics also supports oncologists by interpreting genomic data, identifying treatment options tailored to each patient’s unique molecular profile (20). IBM Watson for Genomics has also demonstrated its effectiveness in personalized treatment through a study in *Oncotarget*, which found that its AI-powered genomic interpretations led to more accurate and individualized treatment options, improving patient outcomes by 25% compared to traditional methods (21). Navican’s AI-driven precision oncology solutions integrate clinical and molecular data to provide personalized treatment recommendations based on the latest medical evidence (22). Navican’s AI-driven precision oncology solutions, can effectively integrated clinical and molecular data to tailor treatment plans, resulting in improved patient outcomes and a 30% increase in therapeutic efficacy compared to standard approaches (23). NantHealth’s platform generates treatment plans by analyzing molecular and clinical data, considering each patient’s genetic makeup, tumor characteristics, and medical history (15, 24). NantHealth’s platform, validated through a study by utilizing molecular and clinical data to create personalized treatment plans, leading to a 25% improvement in patient response rates and a significant reduction in treatment-related adverse effects (17). BenevolentAI leverages AI to analyze biomedical data to identify potential drug candidates and treatment combinations for breast cancer (25). BenevolentAI’s approach, highlighted in a review, which successfully identified new drug candidates and treatment combinations, which led to the discovery of several novel therapeutic options for breast cancer, demonstrating a 40% increase in potential treatment success rates (26). OncoLens uses AI to integrate patient data, treatment guidelines, and clinical evidence to recommend optimal, personalized treatment strategies (27). OncoLens’ AI system, as shown that enhanced personalized treatment strategies by integrating patient data with clinical evidence, resulting in a 20% improvement in clinical decision-making accuracy (28). Deep Genomics specializes in analyzing genetic data to predict the impact of mutations on disease and treatment responses, aiming to identify new therapeutic strategies. Deep Genomics, through research demonstrated its ability to predict the impact of genetic mutations on disease progression and treatment responses, leading to more targeted therapies and a 15% increase in treatment effectiveness (29). Freenome is developing AI-driven blood tests for early cancer detection and personalized treatment monitoring, allowing for real-time adjustments to treatment plans (18). AiCure offers AI-powered solutions to improve medication adherence through computer vision and machine learning, ensuring patients follow their prescribed therapies. AiCure’s AI-powered solutions for medication adherence, detailed in a study shown that it improved adherence rates by 25%, ensuring more consistent therapy and enhanced treatment outcomes (30–33). These advancements highlight the significant role of AI in tailoring breast cancer treatment to improve patient outcomes by addressing individual characteristics and needs (19).

## Regenerative AI in breast cancer drug discovery

Generative AI models are transforming drug discovery for breast cancer by enabling the rapid and efficient creation of novel drug candidates. For example, Insilco Medicine uses generative adversarial networks (GANs) and reinforcement learning algorithms to design new molecules with therapeutic potential (20). Their AI models, trained on extensive databases of chemical compounds and their biological activities, generate virtual libraries of structurally diverse molecules optimized for specific breast cancer drug targets. These molecules are then screened using molecular docking simulations and other computational methods to prioritize candidates for further experimental validation (34). Atomwise utilizes convolutional neural networks (CNNs) to predict the binding affinity of small molecules to specific breast cancer targets. A study demonstrated Atomwise’s success in identifying potential inhibitors for key cancer pathways, with several candidates advancing to preclinical testing and showing promise in animal models (35). Other companies, like Recursion Pharmaceuticals and Numerate, are also harnessing generative AI models to advance breast cancer drug discovery. (22) Recursion Pharmaceuticals applies machine learning to analyze cellular images, identifying compounds that modulate disease-relevant pathways in breast cancer cells. In a study demonstrated that the Recursion’s approach accelerated the identification of effective therapeutic candidates, with several compounds progressing to preclinical studies and showing efficacy in early-stage trials (36, 37). Numerate employs computational chemistry and deep learning to design small molecules with high binding affinity for cancer targets. Their iterative design and screening process, highlighted in a study, which led to the discovery of novel compounds that advanced to preclinical testing, demonstrating high potency and selectivity for breast cancer targets (38). These examples illustrate the diverse applications of generative AI in accelerating drug discovery, ultimately driving the development of more effective and personalized therapies for breast cancer patients.

## Regenerative AI in remote monitoring of breast cancer patients

Remote monitoring tools empowered by regenerative AI are transforming the landscape of breast cancer treatment, offering personalized care and real-time insights for patients and healthcare providers. MyPathway, IBM Watson Health, Breast Cancer Navigator, Tempus, and the Oncology Care Model by Flatiron Health are transforming remote monitoring of breast cancer patients by integrating advanced technologies into current healthcare systems. MyPathway uses a digital platform to provide personalized treatment recommendations based on real-time data from patient health records and clinical trials. This integration facilitates remote monitoring by allowing healthcare providers to track patient progress and adjust treatments accordingly (39).

IBM Watson Health employs AI to analyze vast amounts of medical data, assisting in personalized treatment planning and on-going patient monitoring through its comprehensive data integration and analysis capabilities (40). Breast Cancer Navigator offers a user-friendly interface for patients and clinicians to manage care plans, track symptoms, and schedule follow-ups, thereby improving remote patient management and communication (41). Tempus leverages its platform to provide genomic and clinical data insights, enabling remote monitoring through advanced analytics that support tailored treatment decisions and patient management (42). The Oncology Care Model by Flatiron Health incorporates real-time data collection and analytics to monitor patient outcomes and optimize care coordination remotely (43).

## Challenges of regenerative AI in breast cancer diagnosis and treatment

Regenerative AI, while promising, faces several challenges in the diagnosis and treatment of breast cancer. One significant issue is the limited availability of high-quality, annotated data required for training robust AI models. Study highlights that AI models often struggle with insufficient data, leading to reduced accuracy and generalizability in real-world scenarios (44). Another challenge is the integration of AI tools into existing clinical workflows, which can be disrupted by the technological complexity and need for substantial training. Another study demonstrated that integrating AI into clinical practice requires careful consideration of how these tools fit into current workflows without overwhelming healthcare providers (45). Additionally, there are concerns regarding the interpretability and transparency of AI decisions. Research indicates that the “black-box” nature of AI models presents significant challenges in clinical settings, particularly when it comes to transparency and trust. These models often operate with complex algorithms that are not easily interpretable by human clinicians, making it difficult for them to fully understand how a recommendation or decision is reached. This opacity can create a barrier to trust, as clinicians may be hesitant to rely on AI-generated suggestions without a clear understanding of the underlying reasoning. Consequently, this lack of transparency can impact clinical decision-making, as doctors might question the validity or accuracy of the AI’s advice, leading to potential delays or rejection of useful insights. Moreover, patient trust can also be affected if they perceive that their care is being guided by tools that even their doctors do not fully comprehend. The inability to explain or justify AI-driven decisions can undermine the confidence patients have in their treatment plans, highlighting the need for more interpretable AI models in healthcare (46). Finally, regulatory and ethical issues, such as ensuring data privacy and addressing biases in AI algorithms, pose significant hurdles. A review by Obermeyer et al. discusses how biases in training data can lead to disparities in AI performance across different patient populations, affecting the equity of breast cancer care (47). These challenges must be addressed to fully realize the potential of regenerative AI in enhancing breast cancer diagnosis and treatment. Overcoming the challenges of regenerative AI in breast cancer diagnosis and treatment requires a multi-faceted approach. One effective strategy is improving data quality and availability through collaborative data-sharing initiatives. For example, the Cancer Imaging Archive (TCIA) provides a large repository of annotated medical images, facilitating the development and validation of AI models (48). Ensuring that diverse and representative data sets are used can help address issues of generalizability and bias. Additionally, integrating AI tools into clinical workflows can be optimized by adopting user-centered design principles and providing comprehensive training for healthcare professionals. The study by Esteva et al. highlights that designing AI interfaces to be intuitive and seamlessly incorporated into existing systems can enhance usability and provider acceptance (45). Enhancing AI model interpretability is another crucial strategy. Techniques such as explainable AI (XAI) can make AI decisions more transparent, as demonstrated by Hirides et al., who showed that incorporating interpretability features into AI models can improve clinician trust and decision-making (46). Finally, addressing regulatory and ethical concerns requires rigorous oversight and continuous evaluation. Implementing robust data governance frameworks and conducting regular audits for bias can help ensure that AI tools are equitable and transparent. A review by Obermeyer et al. underscores the importance of addressing these biases through careful scrutiny and adjustment of training data and algorithms to prevent disparities in care (47). By addressing these challenges with targeted strategies, the potential of regenerative AI in breast cancer care can be fully realized, leading to more accurate and personalized treatments.

## Regenerative AI in developing countries

Regenerative AI, characterized by its ability to learn and adapt from new data, holds significant promise for breast cancer diagnosis in developing countries, where healthcare resources are often limited. This technology can enhance diagnostic accuracy and alleviate the burden on overextended medical staff by analyzing mammograms and other imaging studies with high precision. For example, as part of the “Make in India” initiative, Bengaluru-based “Telerad Tech” has introduced “MammoAssist,” an AI-powered tool that detects early-stage breast cancer. It analyzes mammograms and uses a BIRADS Scoring template to categorize findings. Radiologists review the template to confirm and validate results before issuing the final report. Trials show “MammoAssist” boosts radiologist efficiency and productivity by over 50% (49). Another start up named “Niramai Health Analytics” in Bangalore, India, developed an AI-based, low-cost, non-invasive solution for early breast cancer screening in 2016 using body heat mapping which can diagnose breast cancer 5 years earlier than conventional mammography (3). Additionally, regenerative AI continuously improves its diagnostic capabilities by integrating new data, ensuring robust performance in diverse populations in developing country. Another study highlighted the successful implementation of AI-based diagnostic systems in mobile health units in rural India, which improved diagnostic accuracy in breast cancer screenings (3). Furthermore, an AI tool integrated into the healthcare framework in sub-Saharan Africa analyzed digital mammograms and provided instant feedback, significantly accelerating the diagnostic process and enabling quicker interventions (50, 51). These examples underscore the transformative potential of regenerative AI in enhancing breast cancer diagnosis and patient care in developing countries.

## Advantages and disadvantages of the available AI tools

AI tools for regenerative imaging in breast cancer detection offer both promising advantages and notable disadvantages. On the positive side, these tools enhance the accuracy and efficiency of detecting breast cancer by analyzing vast amounts of imaging data that may be beyond human capability. They can identify subtle patterns or early signs of malignancy, potentially leading to earlier diagnosis and improved patient outcomes. AI can also standardize readings, reducing variability between radiologists and leading to more consistent assessments. Additionally, these tools can streamline workflows, allowing for faster analysis and enabling clinicians to focus more on patient care.

However, there are also significant disadvantages to consider. The “black-box” nature of many AI models means that the decision-making process is often opaque, leading to potential mistrust among clinicians and patients. Moreover, AI tools require extensive, high-quality data to function effectively, which may not always be available or may be biased, potentially leading to inaccurate predictions. The integration of these tools into existing healthcare systems can be challenging, requiring substantial resources for training, maintenance, and updates. Additionally, the overreliance on AI could risk de-skilling clinicians or lead to complacency, where human oversight is reduced, and potentially allowing errors to go unnoticed. Therefore, while AI offers significant potential in breast cancer detection, these tools must be used judiciously, with an awareness of their limitations and the need for continuous human involvement.

## The way forward

Addressing the challenges associated with Regenerative AI in breast cancer diagnosis and treatment requires a coordinated approach involving all stakeholders in the healthcare ecosystem, including patients, clinicians, researchers, policymakers, and technology developers.

## Data Availability

The original contributions presented in the study are included in the article/supplementary material. Further inquiries can be directed to the corresponding author/s.
